# Supplementary material for: Exploring Barriers to One Health Antimicrobial Stewardship in Sri Lanka: A Qualitative Study among Healthcare Professionals
Source: Antibiotics (Basel). 2022 Jul 19;11(7):968. doi: 10.3390/antibiotics11070968 (PMC9311535; doi:10.3390/antibiotics11070968)
Supplement: Supplementary file 1 [file antibiotics-11-00968-s001.zip › antibiotics-1799550-supplementary.pdf]

Interview guide

Introduction

- *Introduce yourself and introduce the study and tell what the interview will be for.*
  - This interview is part of an international, collaborative project undertaken by the University of Peradeniya and the Royal Veterinary College, University of London. Information collected from this interview will be used for this project only and will not be shared with anyone for any other purpose; your identity will remain anonymous.
- *Ask permission to record the interview on tape.*

Q1. Can you talk about AMR in your professional life?

- Please use examples of how you found out about AMR and how AMR affect your daily work?
- Have you had any negative experiences with resistance? / Have you ever come a cross case where antibiotics have not been effective in treating?
- What are the factors contributing to AMR?
- How do you see in the future, resistance impacting on your practice?

Q2. AMR is rising in Sri Lanka and what are your thoughts about how it spreads?

- Please use examples of how it can spread between people, animals and the environment?

Q3. How do you think antimicrobial resistance could be reduced or overcome?

- Could you describe the strategies that may be useful to help decrease antibiotic use in Sri Lanka?
- What will be the benefits of implementing an antimicrobial stewardship program in Sri Lanka?
- What are the barriers to successfully implementing programs in Sri Lanka?
- What are your thoughts about the training of healthcare professionals regarding AMR?

Finalizing interview

- *Ask if they have any additional comments on what was said during the interview*
- *Ask the interviewee to introduce themselves including age, the highest level of education and years of service in their job?*
- *Thanking the interviewee for spending their time with you*
